# High-level production of human interleukin-10 fusions in tobacco cell suspension cultures

**DOI:** 10.1111/pbi.12041

**Published:** 2013-01-09

**Authors:** Angelo Kaldis, Adil Ahmad, Alexandra Reid, Brian McGarvey, Jim Brandle, Shengwu Ma, Anthony Jevnikar, Susanne E Kohalmi, Rima Menassa

**Affiliations:** 1 Southern Crop Protection and Food Research Centre, Agriculture and Agri-Food CanadaLondon, ON, Canada; 2 Department of Biology, Western UniversityLondon, ON, Canada; 3 Vineland Research and Innovation CentreVineland Station, ON, Canada; 4 Transplantation Immunology Group, Lawson Health Research InstituteLondon, ON, Canada; 5 Plantigen Inc.London, ON, Canada

**Keywords:** interleukin-10, tobacco BY-2 cells, elastin-like polypeptide, molecular farming, protein fusions, protein bodies

## Abstract

The production of pharmaceutical proteins in plants has made much progress in recent years with the development of transient expression systems, transplastomic technology and humanizing glycosylation patterns in plants. However, the first therapeutic proteins approved for administration to humans and animals were made in plant cell suspensions for reasons of containment, rapid scale-up and lack of toxic contaminants. In this study, we have investigated the production of human interleukin-10 (IL-10) in tobacco BY-2 cell suspension and evaluated the effect of an elastin-like polypeptide tag (ELP) and a green fluorescent protein (GFP) tag on IL-10 accumulation. We report the highest accumulation levels of hIL-10 obtained with any stable plant expression system using the ELP fusion strategy. Although IL-10-ELP has cytokine activity, its activity is reduced compared to unfused IL-10, likely caused by interference of ELP with folding of IL-10. Green fluorescent protein has no effect on IL-10 accumulation, but examining the trafficking of IL-10-GFP over the cell culture cycle revealed fluorescence in the vacuole during the stationary phase of the culture growth cycle. Analysis of isolated vacuoles indicated that GFP alone is found in vacuoles, while the full-size fusion remains in the whole-cell extract. This indicates that GFP is cleaved off prior to its trafficking to the vacuole. On the other hand, IL-10-GFP-ELP remains mostly in the ER and accumulates to high levels. Protein bodies were observed at the end of the culture cycle and are thought to arise as a consequence of high levels of accumulation in the ER.

## Introduction

The need for low-cost and efficient production systems is an important factor in pharmaceutical protein production (Lienard *et al*., 2007). A major limitation for the production of recombinant proteins in prokaryotic and lower eukaryotic systems is the lack of, or incorrect, post-translational modifications. Higher eukaryotic systems such as mammalian cells are restricted by their limited scalability and high cost of production. These limitations, compounded with the increase in demand for complex pharmaceutical proteins, focused interest on plants for the production of recombinant proteins.

Plants offer many advantages over conventional protein expression systems such as low production costs, rapid scalability, the absence of human pathogens and the ability to correctly fold and assemble complex multimeric proteins (Twyman *et al*., 2003). As well, plants have the ability to perform the majority of post-translational modifications needed for biological activity of such proteins. There are, however, potential issues for recombinant protein production in whole plants, including containment of genetically modified plants in the environment, product contamination by mycotoxins, pesticides or endogenous plant secondary metabolites (Hellwig *et al*., 2004) and yield variability due to cultivation conditions and local climate differences (Rybicki, 2010).

Plant cell suspension cultures such as tobacco BY-2 cells constitute an alternative technology for producing pharmaceutical proteins. Plant cell suspensions are inexpensive to culture while maintaining their higher eukaryotic abilities for protein maturation, showing a greater similarity to the native mammalian equivalent with respect to N-glycan structure compared to the same protein produced in bacteria or yeast (Gomord and Faye, 2004). Plant cell suspensions are grown in sterile reactors eliminating the risk of contamination with mycotoxins or pesticides; they synthesize fewer secondary metabolites compared to whole plants (Goossens *et al*., 2003), and their optimal performance is independent of soil quality, climate or season. BY-2 cells are able to rapidly multiply 80- to 100-fold in 1 week, providing the potential for a large-scale, low-cost recombinant protein production platform (Nagata *et al*., 1992). Furthermore, because plant cell suspensions are grown in sterile bioreactors, where containment and batch consistency are guaranteed, proteins produced in plant cell suspensions are more likely to proceed successfully and quickly through the regulatory approval system (Fischer *et al*., 2012). The first plant-made veterinary vaccine to be approved by the USDA Centre for Veterinary biologics in 2006 was the poultry Newcastle disease vaccine produced in tobacco NT-1 cell suspension (Sparrow *et al*., 2007). Very recently, taliglucerase alfa became the first biological drug approved by the US Food and Drug Administration for human use that is manufactured in a modified carrot cell suspension by the company Protalix (Fox, 2012; Maxmen, 2012).

Cell cultures are typically used to secrete proteins to the cell culture medium from which they can be easily purified (Huang and McDonald, 2012; Schinkel *et al*., 2005). However, secreted recombinant proteins can be lost due to adsorption to the culture vessel or to degradation by secreted plant proteases (De Muynck *et al*., 2009; Schiermeyer *et al*., 2005). On the other hand, the plant cell ER contains few proteases and provides a protective, oxidative environment suitable for disulphide bond formation (Ma *et al*., 2003). IL-10 is an anti-inflammatory cytokine that has been found to be effective in suppressing diabetes autoimmunity when combined with the GAD65 autoantigen fused to cholera toxin B subunit (Denes *et al*., 2010). Human, viral and murine IL-10 have been produced in plants (Bortesi *et al*., 2009; Menassa *et al*., 2001, 2004; Morandini *et al*., 2011), with IL-10 accumulating to the highest levels when retained in the ER in transient expression and in transgenic tobacco plants (Bortesi *et al*., 2009; Menassa *et al*., 2001). Viral IL-10 was recently reported to accumulate better when expressed in a tetracycline-inducible expression system in BY-2 cell suspension cultures (Bortesi *et al*., 2012). Also, ER-retained IL-10 fused to an elastin-like polypeptide tag (ELP) accumulates to much higher levels than IL-10 alone, both when transiently expressed or stably transformed in tobacco (Conley *et al*., 2009a; Patel *et al*., 2007).

The purpose of the present study was to examine the suitability of BY-2 cells for high-level production of ER-targeted human IL-10 and IL-10 fusions to green fluorescent protein (GFP) or ELP, to examine the effect of ELP on biological activity of IL-10 and to follow the subcellular trafficking of IL-10 during the cell culture cycle. We found that IL-10-ELP stable transgenic cell lines accumulate the highest levels of IL-10 reported to date in a stable transgenic system, but that ELP reduces the activity of IL-10. We also report that subcellular localization using GFP fusions in BY-2 cells should be performed with caution as GFP is cleaved from the fusion and is trafficked to the vacuole where it accumulates, while IL-10 is lost.

## Results

### BY-2 cells accumulate higher levels of IL-10 than transgenic plants and fusion to ELP greatly improves IL-10 accumulation

**Figure 1:**
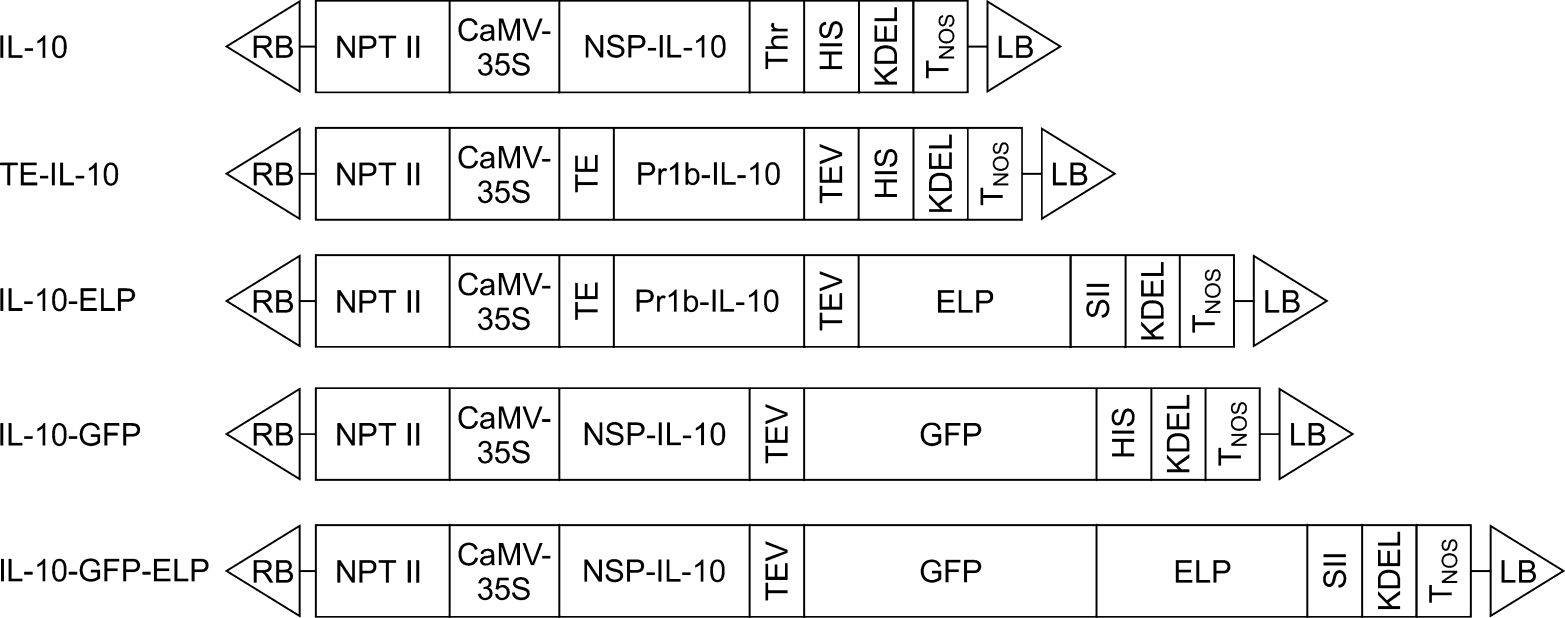
Schematic representation of the constructs used in tobacco BY-2 cell transformation. *NPT II, neomycin phosphotransferase II* gene under the control of the nopaline synthase promoter and terminator; cauliflower mosaic virus (CaMV)-35S, double-enhanced cauliflower mosaic virus 35S promoter. NSP, human IL-10 native signal peptide (54 bp); IL-10, human *IL-10* coding sequence (480 bp); Thr, Thombin protease recognition sequence (27 bp); HIS, six histidine tag; KDEL, ER-retrieval tetrapeptide; T_NOS_, nopaline synthase terminator; tobacco etch virus (TEV), tobacco etch virus protease recognition site (21 bp); green fluorescent protein (GFP), *smGFP* coding sequence (714 bp); TE, translational enhancer from the tCUP promoter (88 bp) (Wu *et al*., 2001); Pr1b, signal peptide from the tobacco *pathogenesis-related 1b* gene (90 bp); ELP, elastin-like polypeptide (420 bp); SII, StrepII purification tag (24 bp); RB, LB, *Agrobacterium tumefaciens* Ti plasmid right border and left border. Constructs are not drawn to scale.

To determine whether BY-2 cell suspensions would be a good system for producing ER-targeted human IL-10, we made several ER-targeted constructs that allowed us both to visualize the subcellular localization of IL-10 as GFP fusions and to increase its accumulation as ELP fusions. The ELP tag consists of 28 repeats of the pentapeptide Val Pro Gly Val Gly derived from the characteristic repeat motif found in native mammalian elastin (Figure S1; Urry, 1988). The constructs consist of IL-10 (Menassa *et al*., 2001), TE-IL-10, IL-10-ELP (Patel *et al*., 2007), IL-10-GFP and IL-10-GFP-ELP (Figure 1).

**Figure 2:**
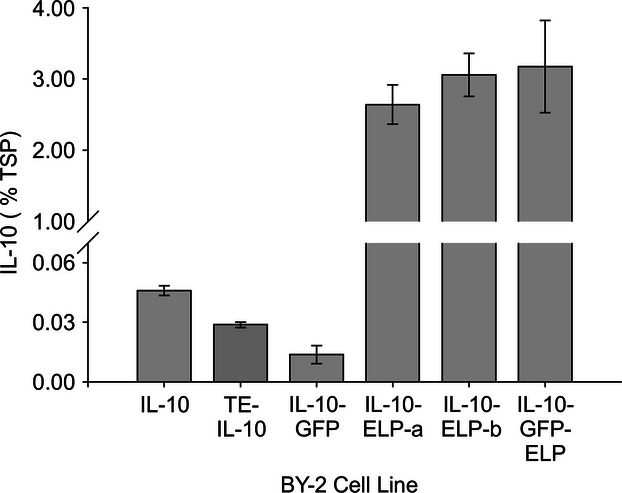
IL-10 accumulation levels in BY-2 cell suspension cultures. IL-10 accumulation levels were determined by ELISA as a percentage of total soluble protein (TSP) in three separate experiments and were averaged. Error bars represent the standard error of the mean of the three experiments. IL-10 levels for TE-IL-10 were averaged from two data points.

Independent transgenic calli were produced for all constructs (12 lines for IL-10, eight lines for TE-IL-10, 18 lines for IL-10-GFP, 50 lines for IL-10-ELP and 34 lines for IL-10-GFP-ELP) and were passed through three rounds of selection, before accumulation levels of IL-10 were determined by ELISA. The highest expressing callus from each population was developed into a cell suspension culture, which was then further analysed. It is noteworthy that accumulation levels of the cell suspensions were several fold higher than the best expressing transgenic plants with the same constructs. For example, the highest IL-10-expressing tobacco stable transgenic plant accumulated 0.0055% total soluble protein (TSP) (Menassa *et al*., 2001), eightfold lower than the highest expressing BY-2 cell suspension (0.046% TSP, 6.42 mg/kg FW, Figure 2). A similar trend was observed with TE-IL-10 and IL-10-ELP (Patel *et al*., 2007; Figure 2). Because the TE-IL-10 line (0.029% TSP, Figure 2) produced lower IL-10 levels than the IL-10 line, we did not use it in further experiments.

The highest expressing IL-10-ELP line accumulated up to 3.057% TSP in cell suspension (average of 762 mg/kg FW, Figure 2). This level is over 500-fold higher than the levels of IL-10 we obtained with stable transgenic plants used in mouse feeding experiments, which resulted in an amelioration of colitis in an IL-10 −/− mouse model (Menassa *et al*., 2007), and the highest level reported to date for human IL-10 produced in a stable plant expression system.

While IL-10-ELP greatly increased recombinant protein accumulation, the addition of GFP did not affect IL-10 levels. The IL-10-GFP line produced 0.0135% TSP (2.28 mg/kg FW), while the IL-10-GFP-ELP line accumulated 3.175% TSP (838 mg/kg FW), similar to the IL-10-ELP lines (Figure 2).

### Effect of transgene copy number and steady-state transcript levels on IL-10 accumulation

**Figure 3:**
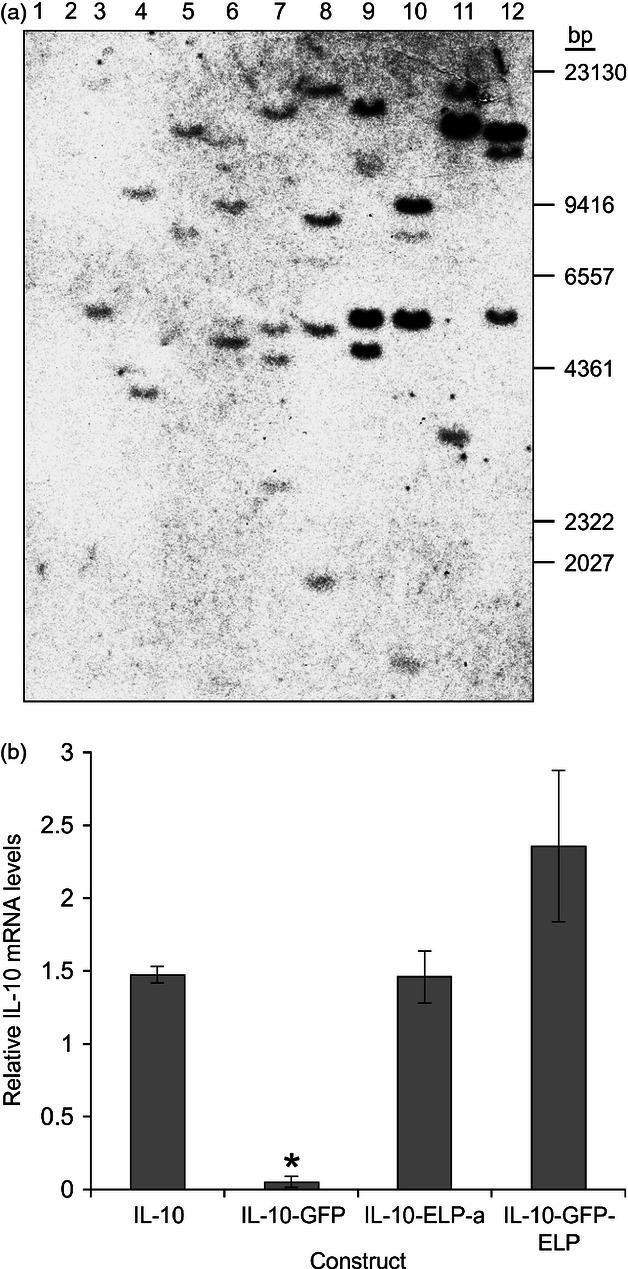
IL-10 gene copy number and steady-state mRNA levels in transgenic BY-2 cell lines. (a) Southern blot analysis of stable transgenic BY-2 cell lines. 10 μg of genomic DNA digested with *Bam*HI (odd numbered lanes) and *Eco*RI (even numbered lanes). Lanes 1 and 2, wild-type BY-2 genomic DNA; lanes 3 and 4, IL-10; lanes 5 and 6, IL-10-ELP-a; lanes 7 and 8, IL-10-ELP-b; lanes 9 and 10, IL-10-GFP; and lanes 11 and 12, IL-10-GFP-ELP. (b) Quantitative RT-PCR was performed to determine the relative IL-10 transcript levels present in transgenic BY-2 cells. IL-10 mRNA levels were expressed as a ratio of actin and α-tubulin reference genes. Significant differences (*P* < 0.05) are indicated (*). Error bars represent the standard error of the mean.

The yield of murine and viral IL-10 was reported to increase in transgenic tobacco between the T_0_ and T_1_ generations (Bortesi *et al*., 2009), which could be due to the doubling of the transgene copy number from hemizygous in the T_0_ to homozygous in the T_1_ generation. Therefore, we determined the transgene copy numbers of five BY-2 cell lines by Southern blotting. For this, genomic DNA was isolated from BY-2 cell cultures after 3 days of growth, and 10 micrograms of genomic DNA was digested with *Bam*HI or *Eco*RI endonucleases (Figure 3a). Each of these enzymes cuts only once either at the 5′ or at the 3′ end of the IL-10 sequence in all constructs. We found that all lines contain multiple copies of the transgene, with the IL-10 line containing two copies of the transgene, IL-10-ELP-a and IL-10-GFP-ELP three copies, IL-10-GFP and IL-10-ELP-b four copies (Figure 3a). The IL-10-ELP-b line was not used in further studies because the cells formed clumps in suspension culture, making it difficult to maintain. All the other lines exhibited growth rates and culture phenotypes similar to wild-type cells.

To find out whether variable transcriptional activity at the transgene insertion sites in the genome may affect mRNA levels and be responsible for differences in recombinant protein accumulation, we determined the steady-state transcript levels for each of four cultured transgenic BY-2 cell lines (IL-10, IL-10-GFP, IL-10-ELP-a and IL-10-GFP-ELP) by quantitative real-time RT-PCR (Figure 3b). Ratios of IL-10 relative to actin transcripts were averaged with ratios of IL-10 to α-tubulin transcripts to determine relative IL-10 expression in each of the four cell lines. This was repeated using three separate cultures for each cell line. The collective data were averaged and analysed for statistical significance (*P* < 0.05) of the differences between the mean relative IL-10 expression values (Figure 3b). Only the IL-10-GFP line showed a significantly lower level of IL-10 steady-state transcript level following statistical analysis. This correlates well with a lower level of protein accumulation and could be due to post-transcriptional gene silencing in this line (Figure 2). There was no significant difference between mRNA levels of the IL-10, IL-10-ELP-a and IL-10-GFP-ELP cell lines, and relative mRNA levels between IL-10 and IL-10-ELP were practically the same. Therefore, the increase in accumulation levels in the IL-10-ELP line over the IL-10 line is not due to the additional transgene copy in the genome or to an increase in the abundance or stability of the transcript. The difference in accumulation is most likely due to a stabilizing effect of ELP on the protein itself, although improved translation efficiency due to the TE element could also have played a role in IL-10-ELP protein levels.

### Nicotine levels in BY-2 cells

Because BY-2 cells are derived from tobacco and previous reports indicated only trace amounts of nicotine and undetectable levels of other alkaloids (Goossens *et al*., 2003), we determined actual nicotine levels in BY-2 cells and compared them to those in leaves of the low alkaloid tobacco cultivar 81V9. Using the selected ion monitoring method, we detected extremely low levels of nicotine, 53 ng/g dried cells. Compared with 0.178 mg/g dried leaves in 81V9 leaves, BY-2 cells contain over 3000-fold lower nicotine levels, making them more suitable for oral administration of either whole cells or partially purified proteins.

### Biological activity of purified IL-10-ELP protein

**Figure 4:**
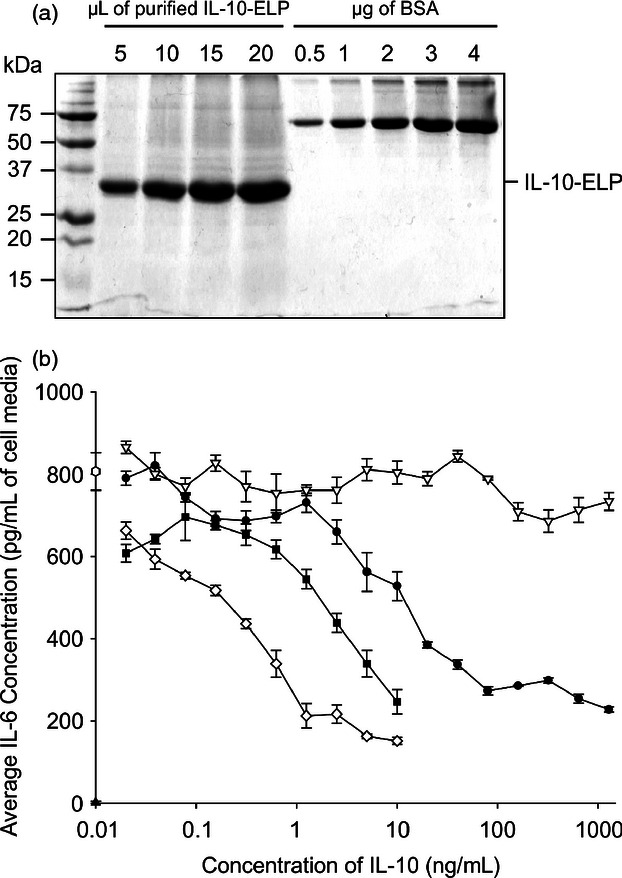
Purification and biological activity of IL-10-ELP. (a) Stained gel of purified IL-10-ELP protein. Lane 1, protein marker; lanes 2–5, increasing volumes of purified IL-10-ELP product; lanes 6–10, increasing amounts of purified, commercial BSA standard. (b) Biological activity analysis of IL-10-ELP. The cell culture supernatant of 4 × 10^5^ PU5-1.8 cells (▲), lipopolysaccharide(LPS)-stimulated cells (◯), LPS-stimulated cells treated with commercial rhIL-10 (◊), IL-10-ELP (•), IL-10 moiety of IL-10-ELP after cleavage of fusion and removal of ELP (▪). An equal volume of sample purified from wild-type BY-2 cells (▽) was used as a negative control.

We have previously reported on the effect of ELP on accumulation levels of several recombinant proteins in transgenic plants and in transient expression in *N. tabacum* leaves (Conley *et al*., 2009a; Patel *et al*., 2007), and we have shown that ELP does not interfere with the binding of erythropoietin with its receptor (Conley *et al*., 2009a). However, IL-10 is a noncovalently-bound homodimer, and we investigated whether ELP might interfere with folding and assembly of IL-10 and therefore affect its biological activity. For this, we purified IL-10-ELP by a two-step procedure taking advantage of the thermally responsive property of ELP enabling a simple nonchromatographic method for protein purification called ‘inverse transition cycling’ (ITC) (Meyer and Chilkoti, 1999). We found previously that ITC does not effectively purify an ELP-tagged protein from plant extracts, rather it helps in concentrating it (Conley *et al*., 2009a). IL-10-ELP was therefore concentrated by one round of ITC, followed by Strep-Tactin affinity chromatography. Its integrity, purity and concentration were determined by SDS-PAGE (Figure 4a). To determine whether IL-10-ELP dimerizes in BY-2 cells similarly to stable transgenic plants (Patel *et al*., 2007), we separated purified IL-10-ELP by a nonreducing native PAGE and analysed it by immunoblotting with an anti-IL-10 antibody. The presence of two main bands on that blot, similarly to the commercial IL-10, suggests that dimerization is occurring (Figure S2). The purified fusion protein was then tested for activity in a murine macrophage–monocyte cell line (PU5-1.8). In this assay, IL-10 is expected to inhibit lipopolysaccharide (LPS)-induced secretion of IL-6 in a dose-dependent manner (Fiorentino *et al*., 1991). We found that IL-10-ELP did inhibit the secretion of IL-6 in a dose-dependent manner (Figure 4b), but at significantly higher concentrations than the rIL-10 control. A comparison of the linear regions of the sigmoidal curves suggests that to achieve the same level of activity as the commercial rIL-10 protein, an approximate average of 80 times more of the IL-10-ELP fusion protein is required. To verify whether ELP was interfering with binding of IL-10 to its receptor, we proteolytically removed ELP using the TEV protease. Removal of the ELP tag from the fusion protein resulted in a 10-fold reduction in the amount of IL-10 needed for dose-dependent inhibition of IL-6 secretion.

### Subcellular localization and characterization of IL-10-GFP and IL-10-GFP-ELP

**Figure 5:**
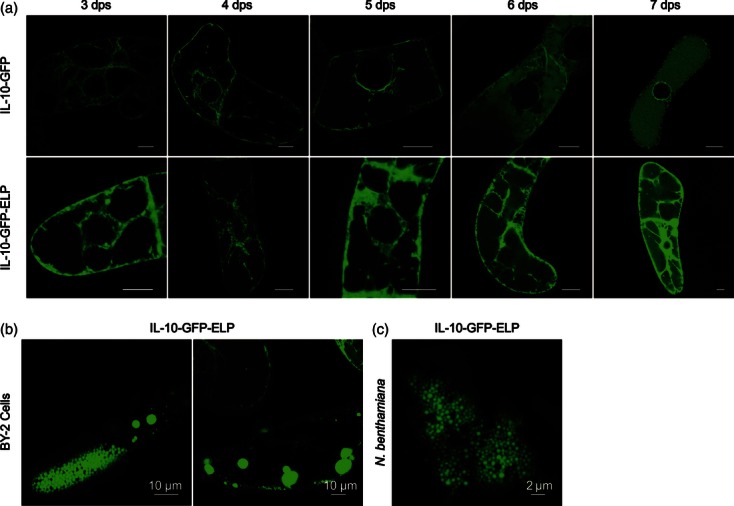
Subcellular localization of IL-10-GFP fusions in BY-2 cells. (a) Time course of tobacco BY-2 cells expressing IL-10-GFP and IL-10-GFP-ELP, visualized by confocal laser scanning microscopy. dps, days postsubculturing. Size bars = 10 μm. (b) IL-10-GFP-ELP produces protein bodies in 10% of BY-2 cells. (c) IL-10-GFP-ELP produces protein bodies in most cells when transiently expressed in *N. benthamiana*.

Because such a large increase in accumulation levels was observed with the ELP tag, the IL-10-GFP and IL-10-GFP-ELP fusion constructs were used to visualize the subcellular localization of the recombinant proteins. These constructs should allow us to determine whether the increased expression levels observed with the ELP fusion are associated with the formation of protein bodies similar to those observed in GFP-ELP transient expression in *N. benthamiana* (Conley *et al*., 2009b). For both constructs, GFP fluorescence showed a reticulated pattern typical of the ER after 3 and 4 days postsubculturing (dps; Figure 5a), when the cells are in their log phase of growth (Matsuoka *et al*., 2004). In addition to the reticulated pattern, a haze of green fluorescence was seen throughout the vacuolar compartments at 6 and 7 dps, during the stationary phase of growth (Matsuoka *et al*., 2004). By 6 dps, the reticulated pattern was barely detectable with IL-10-GFP (Figure 5a), although the overall fluorescence seemed to intensify with each day, suggesting that the recombinant protein was accumulating in the vacuole. On the other hand, for IL-10-GFP-ELP, the ER reticulated pattern was visible throughout the culture cycle, a faint green haze in the vacuole was apparent at 6–7 dps (Figure 5a), and numerous fluorescent protein bodies were observed in 10% of the cells only at 7 dps (Figure 5b). Confocal analysis was repeated twice with a different cell suspension batch, and the same pattern of fluorescent protein bodies was observed at 7 dps.

The IL-10-GFP-ELP construct was also transiently co-expressed in *N. benthamiana* leaves with the p19 suppressor of post-transcriptional gene silencing (Silhavy *et al*., 2002). We found that numerous protein bodies are present in most cells at 4 days postinfiltration (Figure 5c). We also determined the amount of IL-10 produced in this experiment to be 0.76 ± 0.05% TSP, less than in BY-2 cells.

### Analysis of the integrity of the fusions over the cell cycle

**Figure 6:**
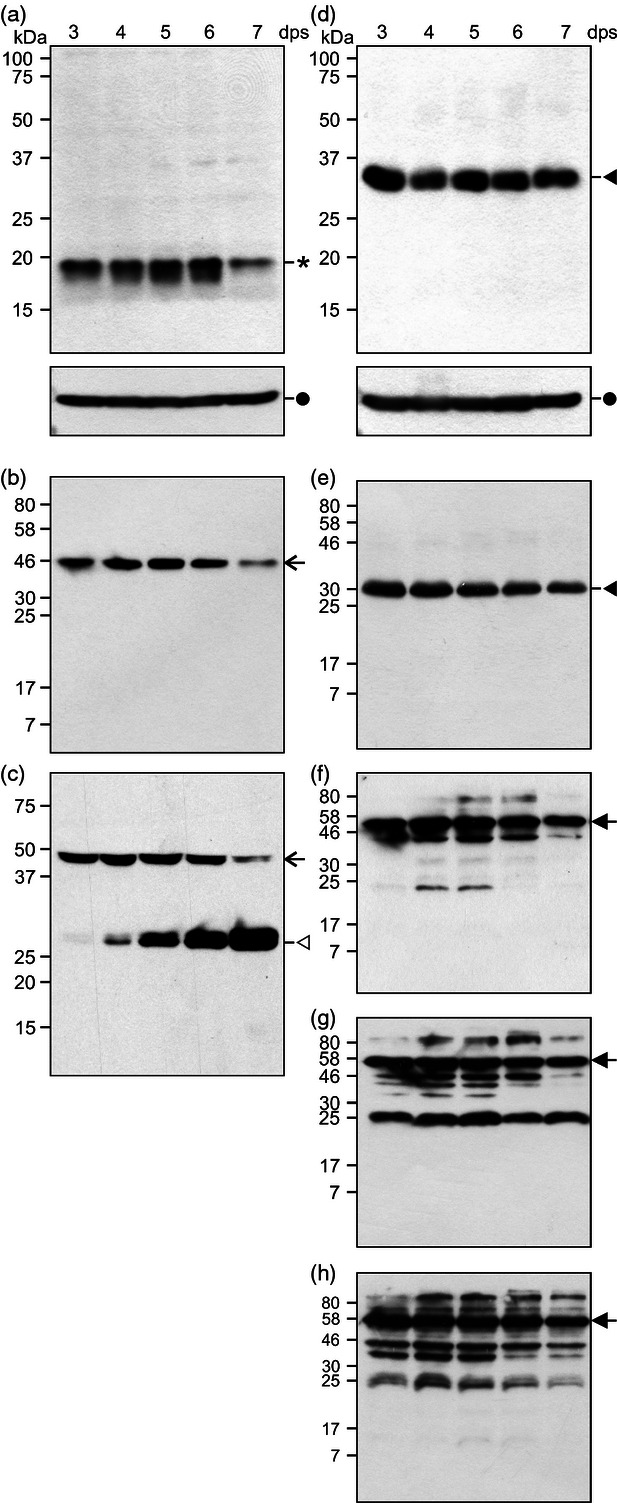
Western blot analysis of the accumulation of IL-10 fusions in tobacco BY-2 cells at 3 to 7 dps. (a) Upper panel is the IL-10 line probed with anti-IL10 antibody; lower panel is the same blot reprobed with anti-α-tubulin antibody; (b,c) IL-10-GFP probed with: (b) anti-IL-10 antibody and (c) anti-GFP antibody; (d,e) IL-10-ELP probed with: (d) anti-IL-10 antibody in upper panel; lower panel is the same blot reprobed with anti-α-tubulin antibody; (e) anti-ELP antibody; (f–h) IL-10-GFP-ELP probed with: (f) anti-IL-10 antibody, (g) anti-GFP antibody and (h) anti-ELP antibody. Asterisk: IL-10, open arrow: IL-10-GFP, open arrowhead: GFP cleavage product, closed arrowhead: IL-10-ELP, closed arrow: IL-10-GFP-ELP, closed circle: α-tubulin. Blots a-c: 75 μg TSP/lane; blots d-h: 1 μg TSP/lane. Molecular weight markers (kDa) are shown on the left; dps, days postsubculturing.

The integrity of recombinant IL-10 and IL-10-GFP was investigated over the cell culture cycle by Western blot analysis. The blots were detected with antibodies against either IL-10 or GFP. For both of these cell lines, the levels of the 19 kDa IL-10 or 47 kDa fusion protein reached its plateau after 4–5 dps, followed by a gradual decline (Figure 6a,b). Upon probing with a GFP antibody, the IL-10-GFP line showed the same pattern of accumulation for the 47 kDa fusion protein, as well as the presence of a ∼27 kDa band, corresponding to the size of the GFP portion of the fusion. This GFP fragment increased in abundance daily and became more abundant than the IL-10-GFP fusion after 5 dps (Figure 6c). As no cleaved IL-10 fragment is detected with the anti-IL-10 antibody, this suggests that IL-10 is degraded beyond recognition.

Because IL-10-ELP and IL-10-GFP-ELP proteins accumulate to much higher levels than the IL-10 or IL-10-GFP proteins and to determine whether fusions with ELP also are cleaved, a similar experiment was conducted with the IL-10-ELP and IL-10-GFP-ELP lines. The IL-10-ELP line showed a single band corresponding to the full-length fusion protein when immunodetected with either an anti-IL-10 or an anti-ELP antibody (Figure 6d,e). No degradation products were detected in any of the days in this cell line, indicating that the fusion remained intact throughout the culture cycle. A complex banding pattern was observed with the IL-10-GFP-ELP cell line. Immunoblotting with anti-IL-10, anti-ELP and anti-GFP antibodies consistently detected a ∼57 kDa protein in all samples, which is the expected size of the full-length fusion protein (Figure 6f–h). However, several other bands were detected with IL-10, GFP and ELP antibodies, indicating proteolytic cleavage of the fusion (Figure 6f–h).

These immunoblots suggest that recombinant IL-10 tagged with ELP, with or without GFP, was more stable over time compared to the corresponding lines without ELP (compare Figure 6a–c with 6d–h). IL-10 accumulation peaked at 3–5 dps but declined sharply at 7 dps in the IL-10 and IL-10-GFP lines, while the IL-10-ELP and IL-10-GFP-ELP lines showed a very slight decline in the full-length fusion protein levels. As well, these immunoblots indicate that cleavage occurs in cell lines transformed with constructs containing GFP, but not with the construct containing only ELP. Because both constructs are practically identical, the cleavage must occur within GFP. These results caution us from using GFP fusions for definitive subcellular localization of proteins in cell suspensions and indicate that more experimental evidence needs to be collected to determine the subcellular localization of a recombinant protein. Indeed, it is possible that fluorescence we observed in the vacuole might be due to cleaved GFP and not to the localization of IL-10-GFP fusions to that compartment (Figure 5a).

**Figure 7:**
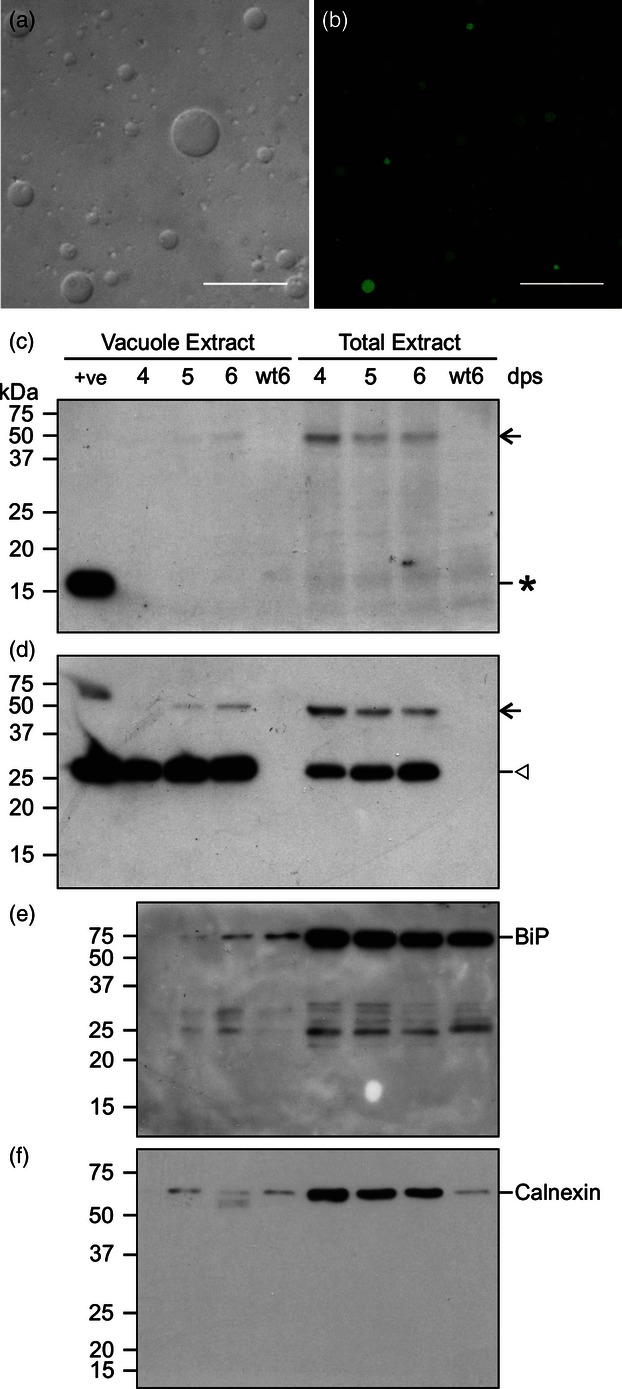
Isolation and analysis of vacuoles from BY-2 cells expressing IL-10-GFP. (a) D.I.C. microscope image of purified vacuoles. (b) GFP fluorescence in purified vacuoles. Scale bars = 30 μm. (c–f) Western blot analysis for IL-10-GFP cell suspension at 4–6 dps. Samples containing 10 μg of protein extracted from purified vacuoles or 10 μg of total soluble protein were immunoblotted with antibodies for: (c) IL-10; (d) GFP; (e) HSC70 (BiP); (f) calnexin. The first lane in panels (c) and (d) (+ve) contains 20 ng of recombinant IL-10 or 20 ng of recombinant GFP, respectively, as positive controls. Asterisk: IL-10, open arrow: IL-10-GFP, open arrowhead: GFP cleavage product. Molecular weight markers, in kilodaltons, are shown on the left; dps, days postsubculturing; wt, wild type.

To determine whether GFP fluorescence in the vacuole is due to the full-length fusion or to cleaved GFP, we isolated intact vacuoles from transgenic BY-2 cells expressing IL-10-GFP at 4 to 6 dps. The integrity of the vacuoles and the absence of any contaminating protoplasts in the final isolates were confirmed by differential interference contrast (DIC) microscopy (Figure 7a). The presence of GFP in vacuole preparations was visualized by confocal microscopy (Figure 7b).

An immunoblot of proteins extracted from purified vacuoles and from whole cells detected with the anti-IL-10 antibody (Figure 7c) revealed a very faint −47 kDa band at 6 dps in vacuolar extracts and a clear band in total cell extracts at 4 dps, which decreased in abundance at 5 and 6 dps. An IL-10 cleavage product could not be detected in the vacuoles or in the total extract. The blot detected with the anti-GFP antibody (Figure 7d) displayed the results seen for the fusion protein more clearly: its accumulation increased in the vacuoles from 4 to 6 dps and conversely became less abundant with time in total protein extracts. On the other hand, much more GFP was present in vacuole extracts than in total extracts. To verify whether the vacuolar extracts were devoid of contamination from other compartments, identical blots were detected with antibodies against heat shock protein 70 (Hsc70, also called BiP, a molecular chaperone localized mainly in the ER, Figure 7e) and calnexin (an ER membrane protein, Figure 7f). Both blots showed trace amounts of BiP and calnexin in isolated vacuole samples, indicating some contamination of the vacuole preparation with ER membrane and ER luminal proteins. Therefore, the fusion protein we observed in the vacuole extracts is most likely due to contamination with ER fraction. We postulate that the fusion is cleaved in the ER and GFP-KDEL is trafficked to the vacuole by an unknown mechanism, as observed by other groups (Gomord *et al*., 1997).

## Discussion

The production of recombinant IL-10 in plants has been reported in a number of studies. Murine IL-10 accumulates to 0.7% TSP in *Arabidopsis* seeds (Morandini *et al*., 2011) and 0.6% TSP in transgenic tobacco leaves, while viral IL-10 accumulates to 0.1% TSP (Bortesi *et al*., 2009) and human IL-10 accumulates to 0.005% TSP in transgenic tobacco leaves (Menassa *et al*., 2001). The lower accumulation levels of human IL-10 may be due to its lack of glycosylation (Menassa *et al*., 2007), while the murine form of the protein is glycosylated both *in vivo* and *in planta*. Viral IL-10 is not glycosylated, and it is thought that lower accumulation levels were due to its toxicity (Bortesi *et al*., 2009, 2012). An inducible expression system in BY-2 cells allowed the accumulation of 3.5-fold more viral IL-10, without reaching the levels of accumulation of murine IL-10 (Bortesi *et al*., 2012). It has been documented that glycosylation can stabilize proteins and hence lead to higher accumulation levels (Conley *et al*., 2009c), and therefore, it is possible that both human and viral IL-10 are turned over more readily because of lack of glycosylation. Our interest lies with producing human IL-10 (hIL-10) that can be tested in murine cell assays and is the therapeutic molecule that ultimately would be advanced to human testing. To test the idea that ELP stabilizes proteins *in vivo* and to visualize trafficking of IL-10, we generated BY-2 lines stably expressing the same ER-targeted human IL-10 construct previously reported (Menassa *et al*., 2001), as well as fusions to ELP and GFP. We found that the highest accumulating IL-10 BY-2 line (0.045% TSP) had eight times higher accumulation than transgenic tobacco leaves (0.0055% TSP) (Menassa *et al*., 2001), and the highest accumulating IL-10-ELP line (3% TSP) had about eight times higher accumulation than transgenic tobacco leaves (0.4% TSP) (Patel *et al*., 2007). This might be due to a different composition of the proteome of BY-2 cells that do not contain photosynthetic chloroplasts and contain very low levels of RuBisCo, the main constituent of total soluble protein content in leaves (Baginsky *et al*., 2004; Duby *et al*., 2010). The reduced total protein complexity is an advantage of BY-2 cells as a production system because purification of recombinant proteins is simplified when compared to leaves.

We also observed that the amount of recombinant protein differs between calli and the respective cell suspensions. All levels seem to increase in suspension, although some lines increase more than others. This poses a problem in terms of identifying the highest accumulating line. Therefore, we suggest that the five highest producing calli be developed into cell suspension and tested. The highest accumulating line can then be retained.

To determine whether low accumulation levels of IL-10 are caused by incorrect trafficking, the spatial and temporal localization of IL-10-GFP fusion was analysed by confocal microscopy. During the logarithmic growth stages of the cells (3–4 dps), the fusion protein was successfully trafficked to the ER. However, the intensity of GFP fluorescence seemed to increase and migrate into the vacuole as cell density increased and the culture went into the stationary phase of growth. It has been previously shown that the ER-retrieval signal HDEL can also act as a C-terminal vacuolar targeting peptide for proteins that escape the ER and that this function is independent from accumulation levels (Gomord *et al*., 1997). It has also been reported that ER resident proteins are transported to the vacuole where they are degraded during the stationary phase of BY-2 cell growth (Tamura *et al*., 2004). A recent study of a γ-gliadin-GFP fusion showed a similar pattern of localization in the ER and the vacuole at 3 and 6 dps, concomitant with the gradual increase in abundance of a GFP cleavage product (Francin-Allami *et al*., 2011). These authors concluded that the fusion was transported to the vacuole where it is cleaved. Our immunoblot results of isolated vacuoles indicate that the IL-10-GFP fusion protein is cleaved in the ER, and while GFP is trafficked to the vacuole and accumulates with time, levels of the fusion protein decrease and the IL-10 portion of the fusion is lost, likely via degradation. As well, because cleavage of the fusion starts at 3 dps, while fluorescence in the vacuole is only clearly visible on 6 and 7 dps, it is possible that trafficking of cleaved GFP to the vacuole may be delayed or that low levels of GFP accumulation in the vacuole may not be detectable; in fact, it has been documented that GFP is labile in the vacuole and gets quickly degraded when exposed to light (within 1 h) due to the acidic environment and the protease activity in the vacuole (Tamura *et al*., 2003). Therefore, a minimum amount of GFP accumulation in the vacuole might be necessary before its fluorescence can be detected.

The addition of a C-terminal ELP_28_ resulted in more than a 66-fold increase in IL-10 levels in BY-2 cells. The 3% of TSP achieved in BY-2 cells is the highest level of IL-10 accumulation reported in any stable plant system. However, we found that although IL-10-ELP fusion protein maintains its cytokine function, a higher dose is required to achieve the activity of commercial rIL-10. Removing the ELP tag resulted in a tenfold increase in activity but did not completely restore IL-10 activity. Re-engineering the IL-10-ELP construct with different linkers could allow better folding of IL-10, thus improving activity of the fusion protein.

To find out whether the ELP tag increases expression levels by packaging the fusion protein into protein bodies that are thought to protect the protein from proteolysis and protect the plant cells from toxicity associated with high levels of expression (Conley *et al*., 2009b, 2011; Joensuu *et al*., 2010), we followed IL-10-GFP-ELP by confocal microscopy throughout the cell culture cycle. We observed protein bodies only at 7 dps and only in 10% of cells, while we observed protein bodies in most cells when the same construct was transiently expressed in *N. benthamiana*. These results need to be investigated further, but they indicate that the primary reason for increased accumulation levels is a stabilization of IL-10 by ELP and that protein bodies arise as a consequence of high accumulation levels in the ER, rather than being the cause for high accumulation levels in BY-2 cells.

## Experimental procedures

### Constructs for IL-10 expression in tobacco BY-2 cells

The ER-targeted IL-10, TE-IL-10 and IL-10-ELP constructs were previously described (Menassa *et al*., 2001; Patel *et al*., 2007). The ER-targeted IL-10-GFP fusion construct was created by first replacing the thrombin cleavage site with a tobacco etch virus (TEV) protease recognition site by PCR, then inserting soluble-modified green fluorescence protein (smGFP; Davis and Vierstra, 1998) with a 6 × histidine tag at the IL-10-TEV C-terminus. The IL-10-GFP-ELP construct was built by a three-way ligation of the following fragments: IL-10-TEV, smGFP and ELPstrepII tag. All constructs contained the ER-retrieval sequence KDEL at the 3′ end and were inserted between the T-DNA border sequences of pCaMterX (Harris and Gleddie, 2001) under the control of the double-enhanced 35S cauliflower mosaic virus (CaMV) promoter (Kay *et al*., 1987) and the nopaline synthase (nos) terminator. All expression constructs were electroporated into *Agrobacterium tumefaciens* strain EHA105 (Hood *et al*., 1993) and used to stably transform tobacco BY-2 cells.

### Stable transformation of BY-2 cells

BY-2 cells were maintained in NT medium (4.3 g/L MS salts; 30 g/L sucrose; 0.5 g/L MES; 1 mg/L thiamine hydrochloride; 0.18 g/L KH_2_PO_4_; 0.243 mg/L 2, 4-D; 0.1 g/L myoinositol; pH = 5.7) in the dark at 26 °C, shaking at 160 X rpm. *Agrobacterium*-mediated stable transformation of these cells was performed according to Alvarez *et al*. (2010). Each transgenic microcallus was passed three times onto NT media supplemented with 50 μg/mL kanamycin and 300 μg/mL timentin. After the third passage, all transgenic calli were analysed for recombinant IL-10 protein accumulation by enzyme-linked immunosorbent assay (ELISA). The highest accumulating line for each construct was maintained as a callus and developed into a liquid suspension culture by resuspending 1- to 2-cm callus pieces in liquid NT medium. Subsequently, transgenic BY-2 lines were maintained by subculturing 5 mL of a 7-day-old culture into 45 mL of NT medium containing 50 μg/mL kanamycin.

### BY-2 cell collection and protein accumulation analysis

Cells from each transgenic BY-2 line were collected on a #2 Whatman filter paper in a Buchner funnel under vacuum. A fresh weight of 60 mg for each sample was frozen in liquid nitrogen and stored at −80 °C. Once all samples had been collected, 200 μL of cold protein extraction buffer (PEB: Phosphate-buffered saline (PBS), pH 7.4; 0.1% Tween-20; 1 mm EDTA; 1 mm PMSF; 1 μg/mL leupeptin; 100 mm sodium L-ascorbate) was added to each tube and sonicated with 20 short bursts from a Sonic Dismembrator (Thermo Fisher Scientific, Pittsburg, PA). The lysates were centrifuged at 20 000 × ***g*** at 4 °C for 10 min, and the cleared supernatants were collected. The amount of total soluble protein (TSP) in each sample was measured using the Protein Assay Reagent (Bio-Rad, Hercules, CA). The concentration of IL-10 protein was determined for each of the transgenic BY-2 cell lines, by comparison with an IL-10 standard curve in an IL-10 ELISA (BD Biosciences, Mississauga, Canada).

For Western blot analysis, all samples were collected over the culture cycle then extracted at the same time, as described above. The TSP in each extract was quantified then separated by 12% SDS-PAGE, and Western blots were processed as described by Conley *et al*. (2009a). The antibodies used are described in supplementary Experimental Procedures (Methods S1) online.

### Isolation of vacuoles from BY-2 cell protoplasts and protein quantification

BY-2 cells were collected from cultures 4 to 6 days postinoculation by filtration then used to isolate protoplasts as described for *Arabidopsis* leaves (Yoo *et al*., 2007). Isolated protoplasts were used to isolate intact vacuoles following a procedure outlined for *Arabidopsis* (Frangne *et al*., 2002). Isolated vacuoles were examined under a microscope to verify the presence of intact vacuoles and the absence of any protoplasts. Aliquots of each preparation were used in a *DC* Protein Assay (Bio-Rad, Hercules, CA) to determine the total protein present in each sample as compared to a bovine serum albumin standard.

### Confocal microscopy

Subcellular localization of the IL-10-GFP and IL-10-GFP-ELP fusions protein was visualized using a Leica TCS SP2 laser scanning confocal microscope (Leica Microsystems, Weltzar, Germany) equipped with a 63 × water immersion objective lens. Excitation was performed with a 488-nm argon laser and a 405-nm UV laser, while GFP fluorescence was detected at 500 to 525 nm.

Transient expression in *N. benthamiana* of IL-10-GFP-ELP and GFP-ELP constructs in conjunction with the p19 suppressor of post-transcriptional gene silencing (Silhavy *et al*., 2002) was performed according to Conley *et al*. (2009b).

### Quantitative RT-PCR on transgenic BY-2 cells

Total RNA was isolated from 100 mg (fresh weight) of cells using the RNeasy Plant Mini Kit (QIAGEN Sciences, Germantown, MD). A complementary DNA template was synthesized with an oligo-(dT) primer and 2.5 μg of total RNA using the SuperScript First Strand Synthesis System (Invitrogen, Carlsbad, CA). Quantitative real-time PCR was conducted on a LightCycler 480 Instrument II (Roche, Mannheim, Germany) with the LightCycler 480 SYBR Green I Master Kit (Roche, Mannheim, Germany). The cycling parameters were 95 °C for 10 min followed by 35 cycles of 95 °C for 15 s, 55 °C for 15 s and 72 °C for 15 s (Table 1). IL-10 mRNA levels were expressed as a ratio of the actin and α-tubulin reference genes. The average ratios of three biological replicates were analysed by SAS v. 9.1 (SAS Institute Inc., Cary, NC) using the MIXED procedure (Tao and Littell, 2002) for statistical significance (*P* < 0.05).

**Table:** Genes and primers used in quantitative real-time RT-PCR

Gene	Primer sets	References
Human IL-10	5′-GTGATGCCCCAAGCTGAGA-3′5′-CACGGCCTTGCTCTTGTTTT-3′	Giulietti *et al*., 2001
Tobacco actin	5′-TGGTATGGGTCAAAAGGATG-3′ 5′-CAGGAGCAACACGCAACT-3′	Blackman and Hardham, 2008
Tobacco α-tubulin	5′-GATGTTGTGCCAAAGGATGTCA-3′ 5′-GGCTGATAGTTGATACCACACTTGAAT-3′	Cortleven *et al*., 2009

### Southern blot analysis for IL-10 copy number in transgenic BY-2 cell lines

Samples of each BY-2 cell line were collected on a #2 Whatman filter paper in a Buchner funnel under vacuum, and 0.5 g of cell fresh weight was frozen with liquid nitrogen and powdered with a mortar and pestle. The CTAB method to isolate genomic DNA was described by Porebski *et al*. (1997). The concentration of genomic DNA was quantified using a Nanodrop 1000 spectrophotometer (Thermo Scientific, Rockford, IL). 10 μg of each genomic DNA sample was separately digested with *Bam*HI and *Eco*RI endonucleases, separated on a 0.8% agarose gel, then depurinated, denatured, neutralized and transferred to a nylon membrane. A digoxigenin (DIG)-labelled probe of the coding sequence of IL-10 was prepared using the DIG High Prime DNA Labeling and Detection Starter Kit II (Roche, Mannheim, Germany), hybridized to the blot and detected with CSPD (Disodium 3-(4-methoxyspiro {1,2-dioxetane-3,2'-(5'-chloro)tricyclo [3.3.1.1^3,7^]decan}-4-yl)phenyl phosphate) according to the manufacturer's instructions.

### Purification of IL-10-ELP

IL-10-ELP was concentrated by ITC by warming the cleared cell lysate to 37 °C and adding NaCl to a final concentration of 3.25 m. The mixture was incubated at 37 °C for 10 min then centrifuged at 12 000 × ***g*** for 10 min at 37 °C. The pellet was resuspended in 1/10 of the original lysate volume with cold PEB. The suspension was clarified by centrifugation at 20 000 × ***g*** for 10 min at 4 °C. IL-10-ELP was further purified by passing the supernatant through a *Strep*-Tactin Macroprep cartridge (IBA, Göttingen, Germany). The integrity, purity and concentration of IL-10 were determined by SDS-PAGE compared to a bovine serum albumin standard after staining with GelCode Blue (Pierce, Rockford, IL).

### Biological activity of IL-10 and removal of ELP fusion

The functional activity of recombinant IL-10-ELP was assessed by its ability to inhibit the secretion of interleukin-6 (IL-6) by a lipopolysaccharide(LPS)-stimulated monocyte/macrophage cell line as described by Menassa *et al*. (2001). Extract from wild-type BY-2 cells was subjected to the purification procedure outlined for IL-10-ELP and used as a negative control in the assay. Commercial recombinant human IL-10 protein was used as a comparative standard (BD Biosciences, Mississauga, Canada). The IL-10-ELP fusion was cleaved with TEV Protease (Invitrogen, Carlsbad, CA) and dialysed into PBS. The histidine-tagged TEV protease and *Strep*II-tagged ELP were removed from the IL-10 sample by incubating the dialysed mixture with 0.25 mL of a 50% slurry of Ni-NTA agarose (QIAGEN Sciences, MD) and 0.25 mL of a 50% slurry of Strep-Tactin Sepharose (IBA, Göttingen, Germany) at 4 °C for 1 h. The suspension was centrifuged at 20 000 × ***g*** for 10 min at 4 °C, and the supernatant containing the cleaved IL-10 protein was collected and quantified by ELISA.

### Analysis of nicotine in BY-2 cells

BY-2 cells (3 dps) and 81V9 tobacco leaves were frozen and freeze-dried. Each sample was then ground to pass through a 2-mm screen using a Wiley mill. Approximately 1.0 g of tissue was weighed, and 10 mL of distilled water, 5 mL of dichloromethane, 5 mL of aqueous 10% sodium hydroxide and 5 mL of dichloromethane containing the internal standard anethole (Sigma-Aldrich, St.Louis, MO) were added. The mixture was shaken for 10 min and centrifuged. An aliquot of the dichloromethane layer was filtered and analysed by GC/MS using a Hewlett-Packard 5890 Series II GC with a model 5971A mass selective detector (ionization voltage 70 ev). The GC system was equipped with a J & W DB-5 capillary column (60 m × 0.25 mm, 0.25 μm film thickness, Agilent Technologies). The GC conditions were as follows: injector temperature, 220 °C; flow rate of carrier gas (helium), 2.4 mL min^−1^. The column temperature was maintained at 50 °C for 0.5 min, then increased at a rate of 5 °C min^−1^ to 125 °C, then 2 °C min^−1^ to 155 °C, held for 8 min and then increased at a rate of 25 °C min^−1^ to 260 °C, then held for 8 min. The MS transfer line temperature was set at 290 °C. Selected ion monitoring (m/z 162) was used for analysis of the nicotine. Electron impact spectra were also recorded. The samples were compared to an authentic standard of nicotine (Sigma-Aldrich, St. Louis, MO).

## Supporting information

**Figure S1** Nucleotide and amino acid sequence of the ELP tag fused to IL-10 and IL-10-GFP. Nucleotides are shown above the line with the corresponding amino acids below.

**Figure S2** Western blot analysis comparing commercial recombinant IL-10 and purified IL-10-ELP protein following separation through a non-reducing, native, 12% polyacrylamide gel. Each lane contains 250 ng of purified protein that yielded similar banding patterns between both proteins when probed with an anti-IL-10 antibody.

**Methods S1** Supplementary experimental procedures.
