# Supplementary figures and images for: High-level production of human interleukin-10 fusions in tobacco cell suspension cultures
Source: Plant Biotechnol J. 2013 Jan 9;11(5):535–45. doi: 10.1111/pbi.12041 (PMC3712471; doi:10.1111/pbi.12041)

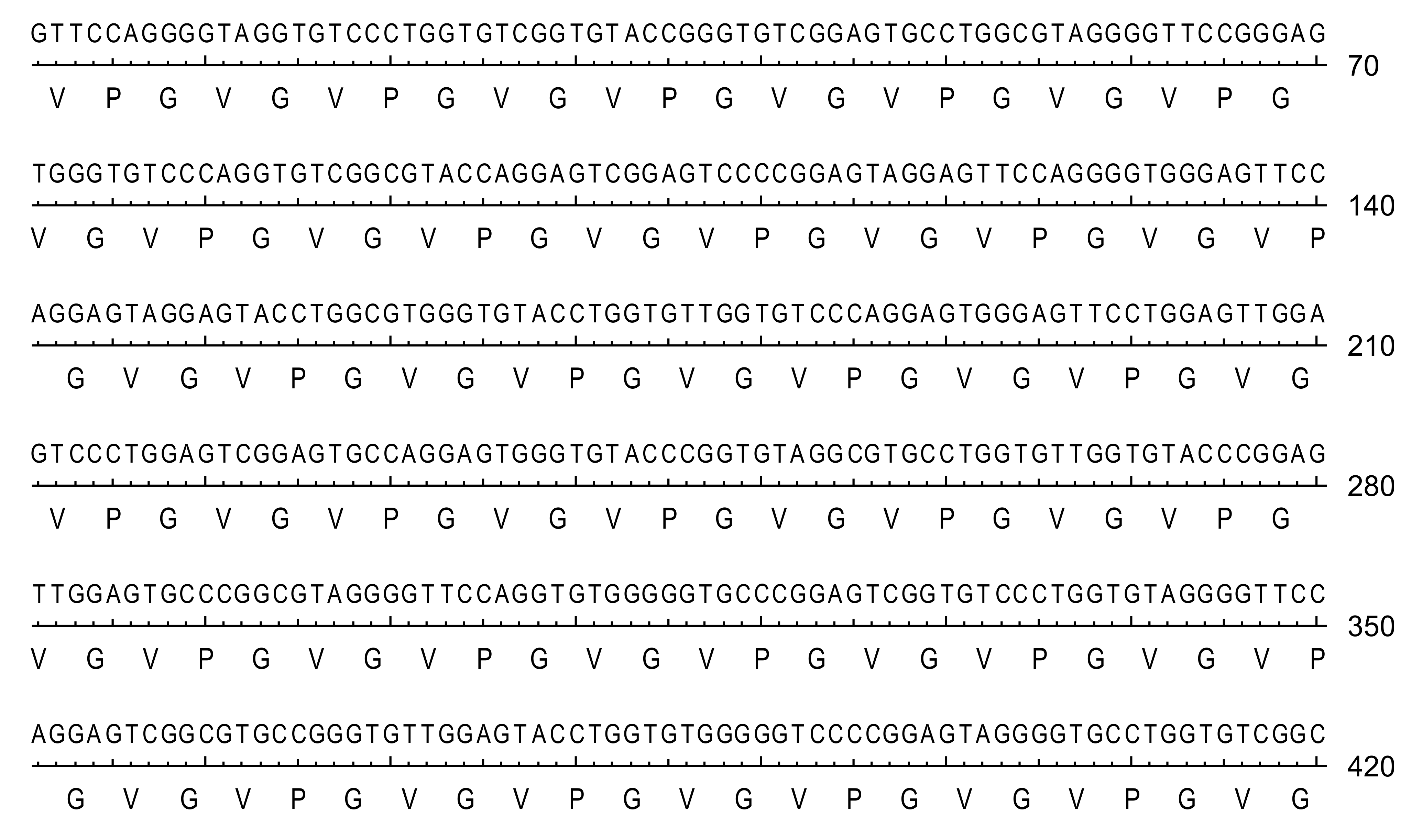

Supplement: Supplementary file 1 [file pbi0011-0535-SD1.tif]

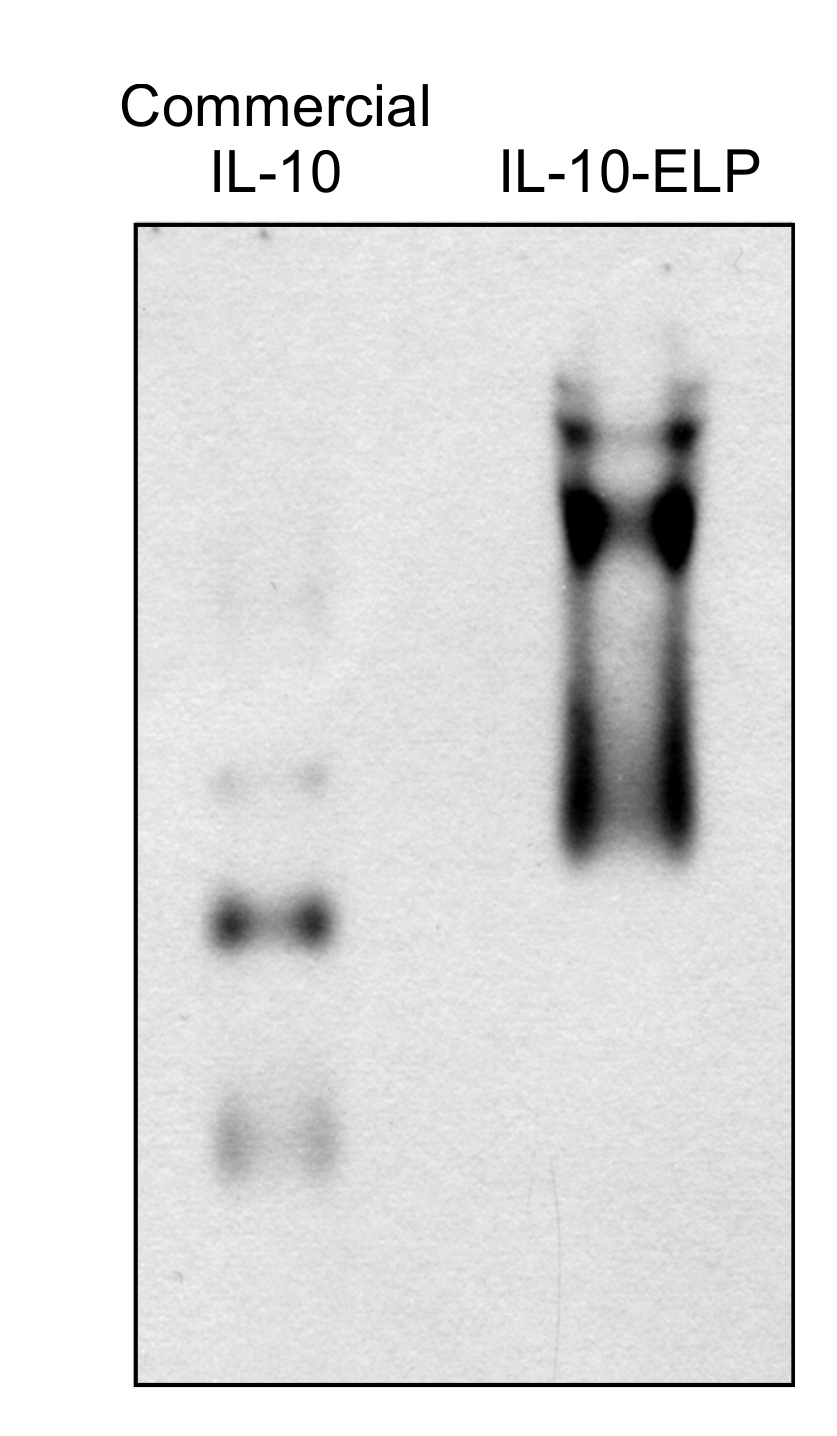

Supplement: Supplementary file 2 [file pbi0011-0535-SD2.tif]
