# Supplementary material for: High-level production of human interleukin-10 fusions in tobacco cell suspension cultures
Source: Plant Biotechnol J. 2013 Jan 9;11(5):535–45. doi: 10.1111/pbi.12041 (PMC3712471; doi:10.1111/pbi.12041)
Supplement: Supplementary file 3 [file pbi0011-0535-SD3.docx]

Supplementary Experimental Procedures:

The antibodies and their dilutions used for western blot detection of the recombinant proteins were a 500-fold dilution of a polyclonal goat anti-human IL-10 (R&D Systems, Minneapolis, USA), detected with a 1000-fold dilution of a polyclonal rabbit anti-goat IgG (Bio-Rad, Hercules, USA); a 1000-fold dilution of a mouse monoclonal Living Colors A.V. (JL-8) (Clontech, Mountain View, USA) (referred to as anti-GFP antibody here), detected with a 3000-fold dilution of a polyclonal goat anti-mouse IgG (Bio-Rad, Hercules, USA), and a 5000-fold dilution of a rabbit antiserum containing anti-ELP ([Patel et al., 2007](#_ENREF_37)), detected with a 5000-fold dilution of a polyclonal goat anti-rabbit IgG (Bio-Rad, Hercules, USA). Control blots showing α-tubulin were the original blots reprobed with 2 μg/ml of a mouse monoclonal anti-α-tubulin antibody (Sigma, St. Louis, USA), detected with a 5000-fold dilution of a polyclonal goat anti-mouse IgG. For BiP and calnexin detection, 1 μg/ml of a mouse monoclonal anti-spinach Hsc70 (Hsp73) (Stressgen, Ann Arbor, USA) antibody was detected with a 1000-fold dilution of a polyclonal goat anti-mouse IgG; and a 100-fold dilution of a monoclonal mouse anti-oat calnexin antibody (gifted by Dr. Heven Sze) was detected with a 3000-fold dilution of a polyclonal goat anti-mouse IgG. All secondary antibodies were conjugated with horseradish peroxidase, so blots were visualized using the ECL Detection Kit (GE Healthcare, Mississauga, Canada). Exposure times to X-ray film varied for all blots. For native immunoblot analysis, separation of 250 ng of purified commercial IL-10 (BD Biosciences, Mississauga, Canada) and BY-2 cell- produced recombinant IL-10-ELP was performed under non-reducing, native conditions by omitting SDS and DTT from the gel buffers and sample buffer prepared for the other western blots above. The blot was probed with a 500-fold dilution of a polyclonal goat anti-human IL-10 antibody, detected with a 1000-fold dilution of a polyclonal rabbit anti-goat IgG and visualized using the ECL Detection Kit.
